# A simple but precise method for quantitative measurement of the quality of the laser focus in a scanning optical microscope

**DOI:** 10.1111/jmi.12249

**Published:** 2015-04-10

**Authors:** J. TRÄGÅRDH, K. MACRAE, C. TRAVIS, R. AMOR, G. NORRIS, S.H. WILSON, G.‐L. OPPO, G. MCCONNELL

**Affiliations:** ^1^ Centre for Biophotonics, Strathclyde Institute for Pharmacy and Biomedical SciencesUniversity of StrathclydeGlasgowU.K; ^2^ Department of PhysicsUniversity of StrathclydeGlasgowU.K

**Keywords:** Beam characterization, knife‐edge measurement, microscopy, resolution

## Abstract

We report a method for characterizing the focussing laser beam exiting the objective in a laser scanning microscope. This method provides the size of the optical focus, the divergence of the beam, the ellipticity and the astigmatism. We use a microscopic‐scale knife edge in the form of a simple transmission electron microscopy grid attached to a glass microscope slide, and a light‐collecting optical fibre and photodiode underneath the specimen. By scanning the laser spot from a reflective to a transmitting part of the grid, a beam profile in the form of an error function can be obtained and by repeating this with the knife edge at different axial positions relative to the beam waist, the divergence and astigmatism of the postobjective laser beam can be obtained. The measured divergence can be used to quantify how much of the full numerical aperture of the lens is used in practice. We present data of the beam radius, beam divergence, ellipticity and astigmatism obtained with low (0.15, 0.7) and high (1.3) numerical aperture lenses and lasers commonly used in confocal and multiphoton laser scanning microscopy. Our knife‐edge method has several advantages over alternative knife‐edge methods used in microscopy including that the knife edge is easy to prepare, that the beam can be characterized also directly under a cover slip, as necessary to reduce spherical aberrations for objectives designed to be used with a cover slip, and it is suitable for use with commercial laser scanning microscopes where access to the laser beam can be limited.

## Lay Description

The resolution of a microscope, that is, how finely detailed the image of your sample is, is determined by the wavelength of the light and the collection angle of the microscope objective. If the objective can collect light leaving the sample in a wide cone, and the excitation light is sent through the objective at the same large angles the resolution should be high. The width of the cone is described by the numerical aperture of the objective. Often, however, the resolution actually achieved in a microscope is worse than expected from the manufacturer's information about the objective. This can be due to aberrations (that is non optimal focussing) or because the input beam is too small (A narrow beam cannot exit the objective at large angles. It is like trying to draw a triangle with short height, narrow base and a large top angle). In order to evaluate the resolution and to find where the problem is, we measure the shape of the excitation beam exiting the objective using a knife edge method. Here a sharp opaque edge is scanned through the beam at different heights and the transmitted light is detected. However, commercially available apparatuses for knife edge measurements are difficult to use under a microscope. Often there is not much space under objective, and you cannot get close enough to the objective with the knife‐edge to study the focus.

In this paper we describe a version of a knife‐edge measurement that works with a microscope. Additionally, the specimen containing the knife‐edge looks (in terms of the amount of glass traversed by the beam) like a real sample so it gives an accurate representation of what the beam would look like in your sample. It overcomes problems with an alternative, commonly used, method to find the resolution based on imaging nanoscale fluorescent beads. There the measured resolution depends on shape of the excitation beam as well as the light detection system and the shape of bead itself, and those three effects can be difficult to separate. Finally, our method is cheap and simple to use.

## Introduction

In microscopy, the resolving power is the most important feature of the optical system and influences the ability to distinguish between fine details of a specimen. According to the Abbe equation, modified by Rayleigh, the lateral resolution *r_lat_* is given by

(1) $$r_{lat}=\frac{0.61\lambda }{N.A.},$$

while the axial resolution *r_ax_* is given by

(2) $$r_{ax}=\frac{2n\lambda }{{\left(N.A.\right)}^{2}},$$

where λ is the wavelength, *n* is the refractive index and N.A. is the numerical aperture of the objective lens (Abbe, 1884; Ditchburn, 1991). These equations are based on the beam profile of a focused plane wave. With reference to Eqs. (1) and (2), for high‐resolution optical microscopy, it is clearly advantageous to use the full numerical aperture of the lens. In laser scanning microscopy, this is usually achieved by overfilling the objective lens. However, most commercial modern microscopes are inaccessible to all but the manufacturers’ service engineers and the user has little or no control over the excitation beam diameter at the back focal plane of the objective lens. Simple and low‐cost methods are needed for the user to evaluate not only the resolution but also the propagation of the laser beam.

The current standard for measuring the resolution of a laser scanning microscope involves excitation of fluorescently labelled beads and then analysing the resultant fluorescence image (Oldenbourg *et al*., 1993; Cox & Sheppard, 2004; Zucker, 2006). This technique gives resolution data through deconvolution of the fluorescence image but since data are only obtained at the point of light–matter interaction, the propagation of the beam is not visualized, and hence information regarding effective numerical aperture of the objective lens is not revealed.

A TEM_00_ beam, which is the beam profile expected for most lasers used for laser scanning microscopy, has a spatial profile that is very nearly Gaussian and the profile does not change as it propagates. The beam divergence θ for an ideal, diffraction‐limited Gaussian laser beam is given by

(3) $$\theta =\frac{\lambda /n}{\pi w_{0}},$$

where *w_0_* is the beam waist radius (where the intensity is 1/e^2^ times the maximum value). The propagation of such an ideal Gaussian beam can be described by

(4) $$w\left(z\right)=w_{0}\sqrt{1+{\left(\frac{\lambda z}{n\pi w_{0}^{2}}\right)}^{2}},$$

where *w*(*z*) is the position‐dependent beam radius and *z* is the distance along the direction of propagation. In practice, however, no laser achieves this theoretically ideal performance level. The divergence expected from a beam focussed by a lens is given by the numerical aperture

(5) N.A.=nsinθ

and a measured divergence lower than this indicates that the full N.A. is not utilized, possibly because the back aperture of the lens is underfilled.

In practice, the propagation of the laser beam is investigated by scanning the beam across an aperture or knife edge that precedes a photodetector. For a Gaussian beam, the measured power as a function of knife‐edge position has the shape of a Gauss error function. From the resultant Gauss error function and according to Siegman *et al*. (1991), the distance between knife‐edge positions corresponding to the values of 10% and 90% transmission of the beam intensity can be multiplied by 1.561 to provide a value for the beam diameter for an ideal Gaussian beam. By performing the knife‐edge measurement at several z‐positions, it is then possible to plot the evolution of the beam radius with respect to propagation distance and obtain the beam divergence along with the beam waist radius.

Although it is possible to measure submicron beam radii on an optical bench (Firester *et al*., 1977; Chapmana *et al*., 2008), performing knife‐edge measurements on a commercial laser scanning microscope is not trivial because of the limited accessibility to the laser beam exiting the objective. This is particularly problematic for short working distance objectives. Work using highly precise Ronchi gratings (Cohen *et al*., 1984; Cannon *et al*., 1986) has shown that micron‐scale measurements can be made in an optical microscope using reflection imaging, but submicron measurements have not been demonstrated using this technique, presumably because of the low resolution of the gratings. For submicron measurements, Schneider & Webb (1981) proposed the use of different methods, including reflection from an aluminium coating on a glass slide and fluorescence excitation of a subresolution bead moving through the focus. However, the reflection method proposed by Schneider and Webb involves the manufacture of a thin film specimen, while the fluorescence measurement involves deconvolution methods to extract the optical focus information. In the work of Marchenko *et al*. (2011), a thin periodic gold nanostructure was used as a knife edge that was scanned across the laser beam to give detailed measurements of highly focused beam radii. However, this method required fabrication of metal nanostructures deposited on a photodiode surface and facilities for fabricating such structures are usually not available in‐house in microscopy laboratories, making the structures difficult to obtain. Xie *et al*. (2013) used a simpler specimen for their double knife edge: a highly polished silicon wafer mounted on top of a photodiode. This device, while potentially offering a knife edge with a very low curvature, is, however, comparatively thick, about 100 microns, thus contributing a significant diffraction loss. Moreover, since this double knife‐edge method cannot be used with a cover slip, or indeed with a water‐dipping objective lens, spherical aberrations may give misleading results for the most commonly used objective lenses for laser scanning microscopy.

We propose here a simpler knife‐edge method to measure the beam divergence, submicron beam waist, ellipticity and astigmatism of the postobjective radiation in a laser scanning microscope which is also compatible with objective lenses requiring a cover slip. We have used an inexpensive and basic but precise specimen comprising a commercially available reflective transmission electron microscopy (TEM) grid attached to a standard glass slide as the knife edge. The beam exiting the objective lens is translated across the knife edge by performing an XT line scan using the in‐built galvanometer mirrors within the commercial laser scanning microscope. We collect the light transmitted through the hole of the TEM grid by placing an optical fibre directly under the glass slide, and relaying the transmitted light to a large area photodetector. We can, using the method described above, measure the radius of the beam leaving the microscope objective lens. From the beam waist and divergence angle, it is then possible to evaluate the effective numerical aperture of the objective lens. Since the method is based on a transmission measurement, it allows separate characterization of the excitation optics, without the effect of the detection optics (most notably the pinhole).

## Experiment

**Figure 1:**
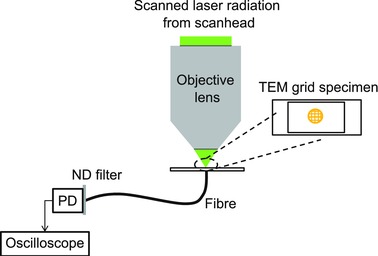
A Leica SP‐5 scanning system (not pictured) directed the scanned radiation to the objective lens. The postobjective radiation was then incident on a specimen comprising a TEM grid mounted on histomount and sandwiched between a type 1.5 cover slip and a standard glass microscope slide. Using high optical zoom, the laser radiation transmitted through a single hole in the grid in XT line scan imaging mode was collected using an optical fibre and sent to a large‐area photodiode connected to an oscilloscope for capture of data.

A schematic diagram of our experimental setup is shown in Figure 1. To evaluate our method, we used a commercial laser scanning microscope (Leica DM6000 upright microscope and SP5 scanning system, Leica Microsystems, Mannheim, Germany). We used an Ar+ laser emitting at 488 nm, and a Ti: Sapphire laser at a wavelength of 800 nm. Objective lenses of 5x/0.15 N.A (HCX PL Fluotar, Leica), 10x/0.4 N.A. (HC PL Apo, Leica), 20x/0.7 N.A. (HC PL Apo, Leica), 40x/0.75 N.A. (HCX PL Fluotar, Leica), and 40x/1.3 N.A (HCX PL Apo, oil immersion, Leica) were chosen to demonstrate the technique.

The fibre used was 1 m long, with a 600 μm core diameter and 0.48 N.A. (M41L01, Thorlabs, Ely, U.K.) and this was held in place by an SMA fibre adaptor plate (CMTSMA, Thorlabs). The adaptor plate was attached to a perspex plate of the same size as a microscope slide, but with a hole in the centre (Chroma Technologies, Bellow Falls, VT, U.S.A.) through which the fibre was fed. The output from the fibre was collected by a Si photodiode (DET100A/M, Thorlabs) with a 50 kΩ resistor connected across the detector output, which was attached to a digital oscilloscope (TDS3242B, Tektronix, Beaverton, OR, U.S.A.) for data capture. The knife‐edge specimen was a copper TEM grid with a square lattice of holes, each 40 μm square (Athene mesh, Agar Scientific, Stansted, U.K.), which was placed on a glass microscope slide. A mounting medium of refractive index approximately 1.5 (Histomount) was applied to the grid specimen, and then a type 1.5 cover slip was added. The mounting medium was allowed to solidify overnight before using the specimen.

To set up the system for measurements of the beam, it was first necessary to align the position of the fibre relative to the objective lens. The laser scanning microscope was set to reflection xyz mode, with low laser power (<100 μW average power after the objective lens for 488 nm, and with <1 mW for 800 nm) and the spectral detection range of the scanning microscope was set to the laser wavelength ±3 nm. The lateral position of the microscope stage holding the fibre in the mount was optimized to obtain a centred reflection image of the fibre core.

**Figure 2:**
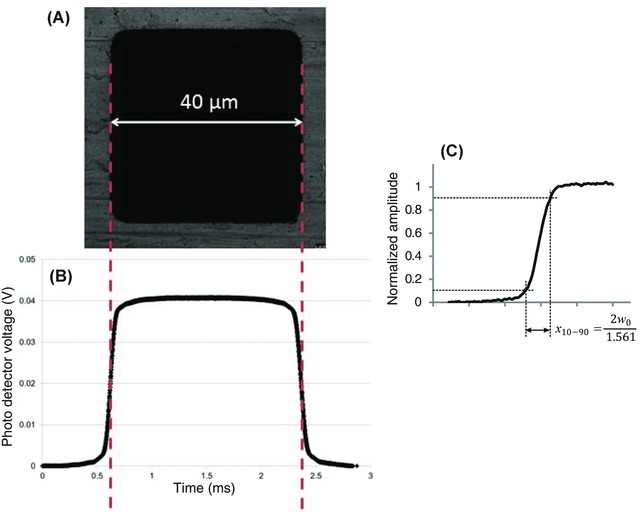
(A) A grey‐scale laser scanning reflection image of a TEM grid with a 40 μm diameter hole, imaged at 488 nm using the 5x/0.15 N.A. objective. (B) The corresponding data acquired using the fibre, photodiode and oscilloscope. This is used to obtain an accurate conversion of the data captured in the time domain on the oscilloscope to the distance measured in the specimen plane. (C) A normalized Gauss error curve obtained for the 5x/0.15 numerical aperture lens, used for measuring the 10% and 90% cut‐off values from which the beam radius w(z) can be obtained.

After this alignment, the knife‐edge specimen was placed directly on top of the fibre and fibre adaptor plate. The axial position of the objective lens was then changed to bring the reflection image of the TEM grid into focus. The TEM grid specimen was manually rotated to be square relative to the field of view. An optical zoom was then applied to bring only one hole of the grid into view, surrounded by around 50% of the width of the metallic grid, as shown in Figure 2(A). Next, the image acquisition mode was changed to provide a line scan across the centre of the square hole, and the line speed set to 10 Hz. This provided a high‐resolution repeating trace on the oscilloscope of the laser radiation transmitted by the hole of the grid relative to the position of the blocking bars (Fig. 2B). This gave a simple means to optimise the axial position of the TEM grid specimen, with the in‐focus specimen providing, as one should expect, the most rapid change from reflected to transmitted light, since the sample is then in the same plane as the beam waist.

Traces were obtained for the specimen in focus, corresponding to the beam waist, and also in the far field, i.e. at least five times beyond the expected Rayleigh range of the beam, by moving the axial position of the objective lens. This provided a range of Gauss error curves, such as the example shown in Figure 2(C). The time interval between the 10% and 90% amplitudes of the error functions was retrieved, and after conversion to distance, the beam radius *w*(*z*) could be measured, as described in the Introduction, and plotted with respect to propagation distance *z*.

In order to obtain the divergence, we measured the actual beam waist and the far field beam diameter *w*(*z*). Using basic trigonometry, the actual beam divergence could be calculated from these values. The beam propagation could be easily compared with the theoretical ideal case, Eq. (4), with the divergence derived from the manufacturer's quoted value of numerical aperture, and the beam waist given by Eq. (3). These measurements were performed for different wavelengths and objective lenses.

The ellipticity and astigmatism in the beam was measured by rotating the scan direction using the microscope controlling software, (Leica LAS AF, Leica). The TEM grid was imaged scanning at angles of 0^o^ and 90^o^, giving line scans perpendicular to each side of the grid square and thus providing data for the *x* and *y* directions of the beam.

## Results

**Figure 3:**
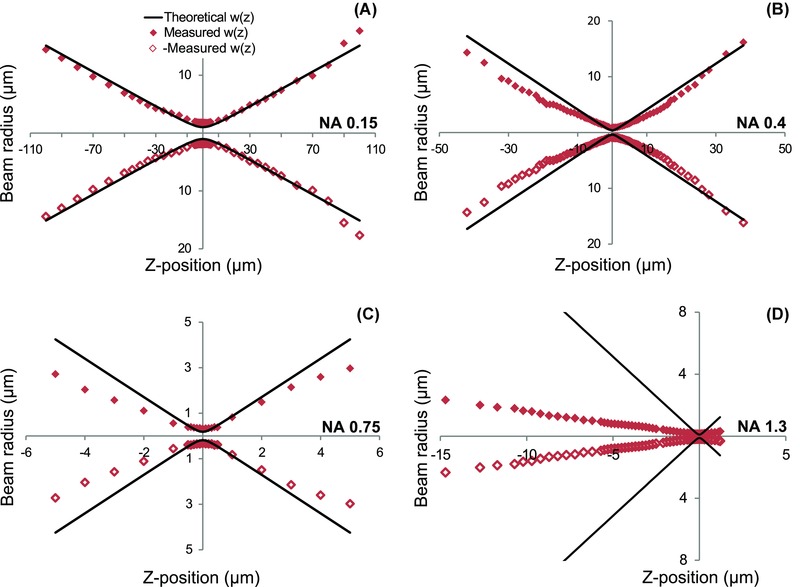
Experimental data of the evolution of the beam radius for the (A) 5x/0.15 N.A., (B) 10x/0.4 N.A., (C) 40x/0.75 N.A., and (D) 40x/1.3 N.A. lens used with a 488 nm laser. The data points (filled diamonds) are presented with the ideal beam propagation for the N.A. of the respective lens. (thick solid line). The data set below the *x*‐axis (open diamonds) is a mirror image of the measured data, to more clearly illustrate the beam propagation.

Using the method described, we could compare our experimental data to the propagation of an ideal Gaussian beam. Figure 3 shows the beam propagation data for the 488 nm line from an Ar+ laser used with the (1) 5x/0.15 N.A., (2) 10x/0.4 N.A., (3) 40x/0.75 N.A. and (4) 40x/1.3 N.A. objective lens. Diamonds represent our data and the thick solid lines are plots of Eq. (4), using the divergence expected from the N.A. of the lens as per Eq. (5). For all these objectives, the beam waist was larger than the theoretically expected value (see also Table 1). For the lower N.A. objectives, the divergence was comparable to the theoretically expected value, and the effective N.A. was close to the N.A. specified for the objective (see Table 1). This combination of a similar divergence and a larger spot indicates that the beam was not brought to a diffraction limited focus, *cf*. Eq. (3). In contrast, the 40x/1.3 N.A. objective had a quite small effective N.A. Images of fluorescent beads taken using 488 nm excitation and the 20x/0.7 N.A. objective (Movie S1 and Fig. S1) had a width in agreement with the beam radius measured using our knife‐edge method, confirming that the larger than theoretically expected beam radius is not an artefact of the method. This also holds for the 40x/0.75 N.A. and 40x/1.3 N.A. objectives (Movies S2 and S3 and Figs. S2 and S3), illustrating that the method gives a reasonable value for the overall beam radius, even for objective lenses, where the paraxial approximation is not strictly valid.

**Table 1:** Measured beam waists and effective N.A. for different objective lenses and wavelengths commonly used in confocal and multiphoton laser scanning microscopy

Objective lens	Wavelength (nm)	Beam waist (nm)	Theoretical beam waist (nm)	Effective N. A.
5 × (0.15 N.A.)	488	1840 ± 38	1030	0.141 ± 0.002
10 × (0.4 N.A.)	488	900 ± 61	378	0.309 ± 0.009
20 × (0.7 N.A.)	488	439 ± 4	200	0.356 ± 0.002
20 × (0.7 N.A.)	800	556 ± 8	328	0.395 ± 0.003
40 × (0.75 N.A.)	488	327 ± 11	183	0.47 ± 0.02
40 × (1.3 N.A.) oil	488	169 ± 2	100	0.291 ± 0.006
40 × (1.3 N.A.) oil	800	265 ± 5	163	0.32 ± 0.03

Note

The theoretical beam waist values are calculated from Eq. (3) and assume use of the full N.A. with appropriate immersion media. The effective N.A. is calculated from Eq. (5), using the measured divergence. The errors in the beam waste are the standard deviation of measurements at three positions very close to the focus.

Table 1 presents an overview of the beam waist measurements for a range of objective lenses and wavelengths used in confocal and multiphoton laser scanning microscopy. Using the highest N.A. lens, it was possible to measure a beam waist as small as 169 nm. The errors are the standard deviation of measurements at three positions very close to the focus. The effective N.A. was calculated from the measured divergence.

The beam size at the entrance pupil of the objective is large enough to fill all objective lenses, save the 10X 0.4 N.A. objective, which is slightly under‐filled. The beam profile is, however, neither flat (approximating a plane wave) nor a truncated Gaussian, and this could have a negative impact on the quality of the focus.

**Figure 4:**
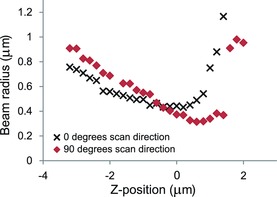
Direct measurement of the ellipticity and astigmatism for a 20x/0.7 N.A. objective lens at a laser wavelength of 488 nm. The figure shows the beam radius with respect to propagation distance *z* measured at 0^o^ rotation of the scan direction (crosses) and at 90^o^ degrees (diamonds).

Beam propagation data to measure ellipticity and astigmatism for a wavelength of 488 nm using a 20x/0.7 N.A. objective lens are presented in Figure 4. Ellipticity in the focused beam was evident through the evolution of the beam radius at 0^o^ and 90^o^. The measured beam waists for each axis of the beam were 440 ± 4 nm and 315 ± 6 nm at 0^o^ and 90^o^, respectively, yielding an ellipticity of 0.72 ± 0.02. This observed ellipticity of the beam is possibly introduced by the scanning system and the prism‐based scan rotation. With regards to astigmatism, the two foci were separated by 1 ± 0.1 μm.

## Discussion

For all the objectives used here, the beam waist was substantially larger than the theoretical value. This could be due to under filling the objective, thus using less than the full N.A., or that the beam is not brought to a diffraction limited focus due to aberrations in the objective lens itself or the beam conditioning optics in the microscope. This will have a negative impact on the achievable resolution in an acquired image. For example, with the 20x/0.7 N.A. objective, which is a convenient choice for both confocal and multiphoton laser scanning microscopy, the beam waist was nearly twice as large as expected at both 488 nm and 800 nm. This would have a substantial impact not only on the lateral resolution, but also on the sectioning capability. Also, the measured astigmatism (shown in Fig. 4) negatively impacts on the confocal range of the source, and reduces the effectiveness of the source for excitation of fluorescence.

The errors in Table 1 are a measure of the noise in the measurement, for example, due to the intensity noise of the laser of about 1%. The error in the measurement can be small in comparison to a standard knife‐edge measurement, since the movement of the scanning galvanometer mirrors and axial movement of the objective lens are accurate and stable. Furthermore, a suitably rapid scan speed can be chosen to overcome any problems with vibration at subkilohertz frequencies, which often reduces the precision of measurements with fluorescent beads or gold nanoparticles (Müller *et al*., 2003).

In choosing the conversion factor between the 10/90 width of the error function and the beam radius, we have assumed in our method that the beam profile is Gaussian, which is in reasonable agreement with the shape of the measured error function traces. Small differences could be due to, for example, spherical aberrations. Beams of other shapes than Gaussian can be analyzed by this knife‐edge type method by choosing appropriate clip values and conversion factors (Siegman *et al*., 1991), since this method makes no assumptions about the beam shape.

We have used the paraxial approximation throughout our analysis, but we have also considered the effects of nonparaxial focusing with low and high N.A. lenses. From the work by Nemoto (1990), the quantity used to measure the degree of deviation from Gaussian propagation is *kw_0_*/√2, where k is the wavenumber. For *kw_0_*/√2 >4, the paraxial approximation is perfectly valid, but for values of *kw_0_*/√2 smaller than this, the optical electric field increasingly deviates from the paraxial approximation. For *kw_0_*/√2 <1, the paraxial approximation completely fails. For the 5x/0.15 N.A. lens, *kw_0_*/√2 is larger than 10 and thus the paraxial approximation is valid. For the 10x/0.4 N.A. lens, *kw_0_*/√2 = 3.4, and the paraxial approximation is reasonably valid. For the higher N.A. objectives with N.A. = 0.7 and N.A. = 1.3, values for *kw_0_*/√2 of 1.82 and 1.38 can be reached for a theoretically minimal beam waist, implying that the paraxial approximation needs corrections to accurately describe the beam propagation. According to Nemoto (1990), the error in the optical electric field can be on the order of 10%. However, Kang & Lü (2005) calculate the deviations from the paraxial approximation of the far‐field divergence and *w_0_*. From this, *w_0_* is less than 10% larger for both objectives. For the objective lens with N.A. = 0.7, the divergence has a minimal deviation from what is expected from the paraxial approximation. For the objective lens with N.A. = 1.3, however, the divergence could be substantially less than that expected from the paraxial approximation, at least for the theoretically minimal beam waist. Experimentally, we also observe more deviations from the paraxial approximation for the objective with N.A. = 1.3. We also note that Eq. (3) assumes that the divergence angle is small, which is not the case for higher N.A. objectives. A complete description of the focussing beam for high N.A. objectives would also need to take into account the beam profile at the entrance pupil of the objective, which for this microscope is not similar to a plane wave. We therefore appreciate that a nonparaxial analysis of the beam propagation may yield slightly different values to those we present here, but it will not change the general observations presented.

The N.A. of the light collecting fibre (0.48) was smaller than the N.A. of the higher N.A. objectives. The associated loss in light collection efficiency of the high‐angle components will lead to a retrieved beam radius that is larger than the actual beam radius (Firester *et al*., 1977), and this effect is more pronounced for higher N.A. objectives. Firester *et al*., however, calculate this error to be about 10% for a collection N.A. as small as one‐third of the N.A. of the beam, assuming an objective N.A. of 0.9. Furthermore, since the *effective* N.A. of all objectives is smaller than the N.A. of the collection fibre, these errors are likely minimal. Even in the unlikely scenario of collecting only the on‐axis light, the error is about 15% (Firester *et al*., 1977).

For higher N.A. objectives, it could be necessary to consider the interaction of the metal knife edge with the electric field of the light, in line with Marchenko *et al*. (2011). Again, these errors are less than about 10%, and if the exact shape of the knife edge is known, these can be corrected for in the analysis (Huber *et al*., 2013). The knife edge should ideally be infinitely sharp on the scale of the beam diameter, but a somewhat graded transmission such as that in our real knife edge would only result in a slight overestimation of the beam radius (Cannon *et al*., 1986). We also note that for the 1.3 N.A. objective, diffraction from the knife edge is visible as a small but abrupt change in the beam diameter and a slightly different divergence after the focus (see Fig. S4).

Finally, the use of the TEM grid specimen rather than complex gold‐patterned slides (Marchenko *et al*., 2011) makes for a simple and inexpensive test target with a high optical damage threshold which does not require careful handling. In view of recent interest in regenerative amplifier lasers for multiphoton microscopy (Mittmann *et al*., 2011; Wang *et al*., 2011), we suggest that the high pulse energies available from these systems may be suitable with the materials and methods used here. With the TEM grid mounted in Histomount under a cover slip, it is also optically very similar to real samples imaged with the microscope, and should therefore give a reasonable representation of the spot size, without introducing further aberrations. If the knife‐edge specimen is prepared with a TEM grid mounted on a slide without a cover slip, it may be possible to measure the properties of the beam for a water‐dipping objective lens.

## Conclusion

We have reported a useful, low‐cost knife‐edge method for accurately measuring the size of the optical focus, beam divergence and ellipticity of the postobjective optical radiation in a laser scanning microscope. Our method can be performed at laser wavelengths currently used in both confocal and multiphoton laser scanning microscopy because of the broadband reflectivity of the copper TEM grid, and at power levels used in optical microscopy since no damage to the specimen results. The method is also suitable for use with microscopes where there is limited access to the laser beam. This approach is likely to be a useful tool in both the routine maintenance of laser scanning microscopes and in their development.

## Supporting information

Disclaimer: Supplementary materials have been peer‐reviewed but not copyedited.

**Fig. S1**. A cross‐section through an in‐focus fluorescent bead (thick green line) taken from Movie S1, a Gaussian with *w_0_* = 0.35 μm (thick dashed line) and an Airy function with full width at half maximum of 0.41 μm (thin black line). The figure illustrates that the beam radius *w_0_* measured with our knife‐edge method (0.439 μm at 0° and 0.315 μm at 90° scan angle) is in reasonable agreement with the beam radius derived from measurements on fluorescent beads. The images in Movie S1 were acquired using 488 nm excitation and the 20x/0.7 N.A. objective.
**Fig. S2**. A cross‐section through an in‐focus fluorescent bead (thick green line) taken from Movie S2 and a Gaussian with *w_0_* = 0.34 μm (thick dashed line). This width corresponds to an illumination spot size of w_0_ = 295 nm convoluted with the fluorescent bead (diameter 200 nm). The figure illustrates that the beam radius *w_0_* measured with our knife‐edge method (327 ± 11 nm) is in reasonable agreement with the beam radius derived from measurements on fluorescent beads. The images in Movie S2 were acquired using 488 nm excitation and the 40x/0.75 N.A. objective.
**Fig. S3**. A cross‐section through an in‐focus fluorescent bead (thick green line) taken from Movie S3 and a Gaussian with *w_0_* = 0.246 μm (thick dashed line). This width corresponds to an illumination spot size of w_0_ = 178 nm convoluted with the fluorescent bead (diameter 200 nm). The figure illustrates that the beam radius *w_0_* measured with our knife‐edge method (169 ± 2 nm) is in reasonable agreement with the beam radius derived from measurements on fluorescent beads. The images in Movie S2 were acquired using 488 nm excitation and the 40x/1.3 N.A. objective.
**Fig. S4**. Experimental data of the evolution of the beam radius for the 40x/1.3 N.A., lens used with a 800 nm laser. The data points (filled diamonds) are presented with the ideal beam propagation for the N.A. of the lens. (thick solid line). The data set below the *x*‐axis (open diamonds) is a mirror image of the measured data, to more clearly illustrate the beam propagation. The data illustrate the effect of diffraction from the knife edge, which results in a small but abrupt change in the beam diameter and a slightly different divergence after the focus.

**Movie S1**. A Z‐stack of images of 200 nm fluorescent beads (Fluoresbrite, Polysciences Inc., Warrington, PA, USA) mounted in Vectashield acquired using 488 nm excitation, a detection range of 500–580 nm and the 20x/0.7 N.A. objective. The images are separated with 100 nm in the *z*‐direction. The astigmatism is clearly visible as the sample is moved through the focus.

**Movie S2**. A Z‐stack of images of 200 nm fluorescent beads (Fluoresbrite, Polysciences Inc.) mounted in Vectashield acquired using 488 nm excitation, a detection range of 500–570 nm and the 40x/0.75 N.A. objective. The images are separated with 340 nm in the *z*‐direction.

**Movie S3**. A Z‐stack of images of 200 nm fluorescent beads (Fluoresbrite, Polysciences Inc.) mounted in Vectashield acquired using 488 nm excitation, a detection range of 500–570 nm and the 40x/1.3 N.A. objective. The images are separated with 170 nm in the *z*‐direction. The astigmatism is clearly visible as the sample is moved through the focus.
