# Supplementary material for: A simple but precise method for quantitative measurement of the quality of the laser focus in a scanning optical microscope
Source: J Microsc. 2015 Apr 10;259(1):66–73. doi: 10.1111/jmi.12249 (PMC4975716; doi:10.1111/jmi.12249)
Supplement: Supplementary file 1 — Fig. S1. A cross‐section through an in‐focus fluorescent bead (thick green line) taken from Movie S1, a Gaussian with w0 = 0.35 μm (thick dashed line) and an Airy function with full width at half maximum of 0.41 μm (thin black line). The figure illustrates that the beam radius w0 measured with our knife‐edge method (0.439 μm at 0° and 0.315 μm at 90° scan angle) is in reasonable agreement with the beam radius derived from measurements on fluorescent beads. The images in Movie S1 were acquired using 488 nm excitation and the 20x/0.7 N.A. objective. Fig. S2. A cross‐section through an in‐focus fluorescent bead (thick green line) taken from Movie S2 and a Gaussian with w0 = 0.34 μm (thick dashed line). This width corresponds to an illumination spot size of w0 = 295 nm convoluted with the fluorescent bead (diameter 200 nm). The figure illustrates that the beam radius w0 measured with our knife‐edge method (327 ± 11 nm) is in reasonable agreement with the beam radius derived from measurements on fluorescent beads. The images in Movie S2 were acquired using 488 nm excitation and the 40x/0.75 N.A. objective. Fig. S3. A cross‐section through an in‐focus fluorescent bead (thick green line) taken from Movie S3 and a Gaussian with w0 = 0.246 μm (thick dashed line). This width corresponds to an illumination spot size of w0 = 178 nm convoluted with the fluorescent bead (diameter 200 nm). The figure illustrates that the beam radius w0 measured with our knife‐edge method (169 ± 2 nm) is in reasonable agreement with the beam radius derived from measurements on fluorescent beads. The images in Movie S2 were acquired using 488 nm excitation and the 40x/1.3 N.A. objective. Fig. S4. Experimental data of the evolution of the beam radius for the 40x/1.3 N.A., lens used with a 800 nm laser. The data points (filled diamonds) are presented with the ideal beam propagation for the N.A. of the lens. (thick solid line). The data set below the x‐axis (open diamonds) is a mirror image of the meas [file JMI-259-66-s001.docx]

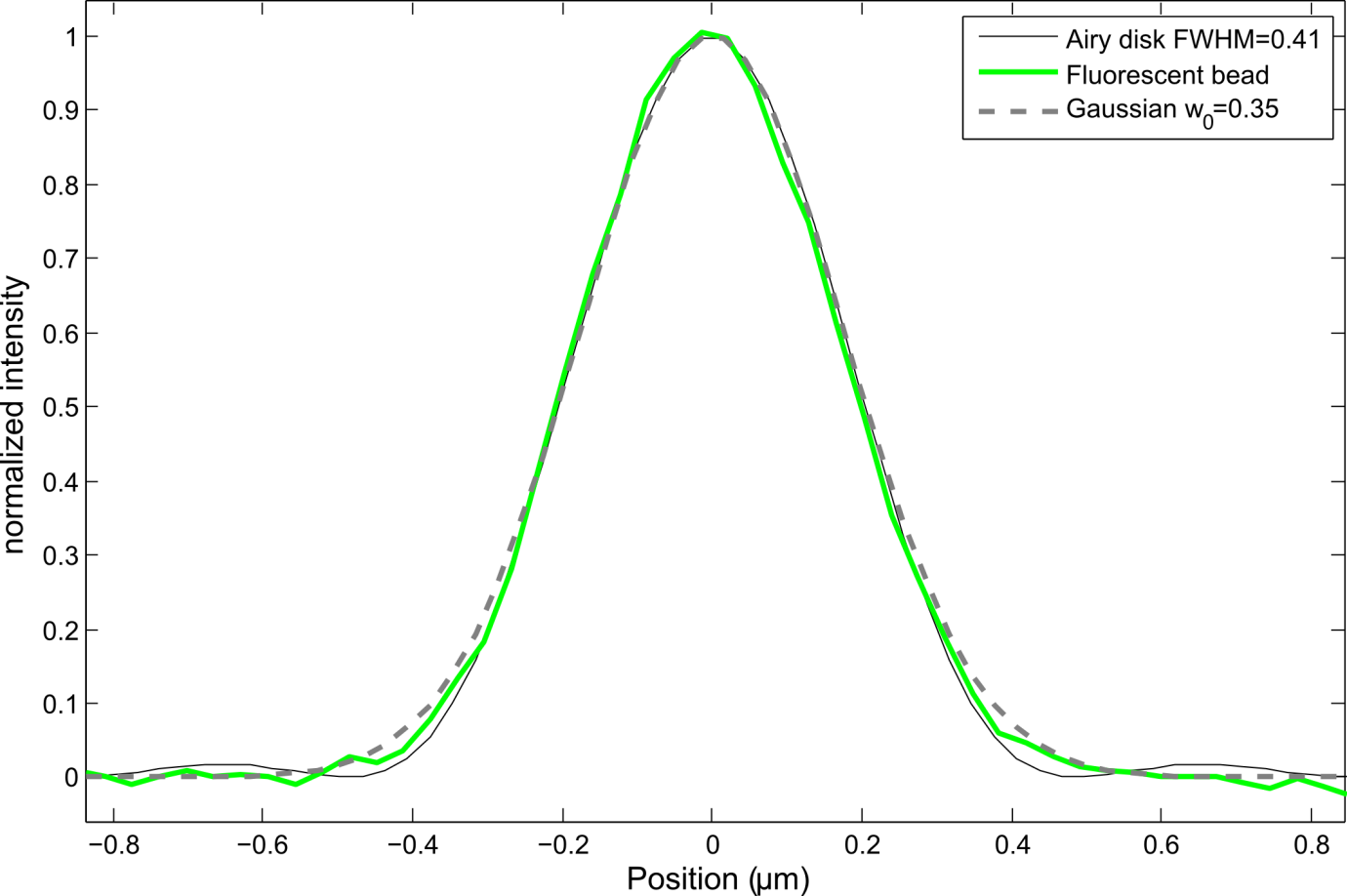


FIG. S1: A cross section through an in focus fluorescent bead (thick green line) taken from Supplementary Movie 1, a Gaussian with *w_0_*=0.35 μm (thick dashed line) and an Airy function with full width at half maximum of 0.41 μm (thin black line). The figure illustrates that the beam radius *w_0_* measured with our knife edge method (0.439 μm at 0 degrees scan angle and 0.315 μm at 90 degrees scan angle) is in reasonable agreement with the beam radius derived from measurements on fluorescent beads. The images in Supplementary Movie 1 were acquired using 488 nm excitation and the 20x/0.7 N.A. objective.


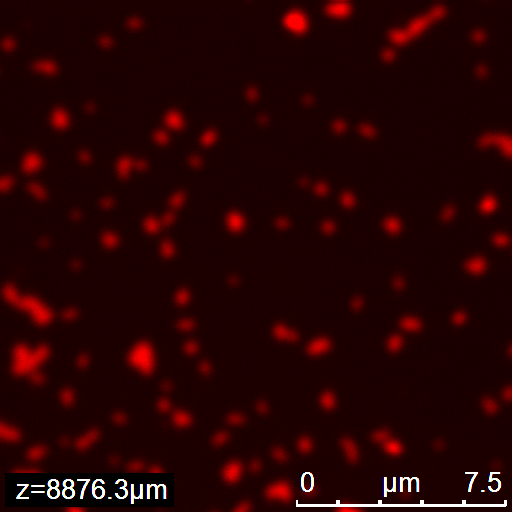


Supplementary Movie 1: A Z-stack of images of 200 nm fluorescent beads (Fluoresbrite, Polysciences inc.) mounted in Vectashield acquired using 488 nm excitation, a detection range of 500-580 nm and the 20x/0.7 N.A. objective. The images are separated with 100 nm in the Z-direction. The astigmatism is clearly visible as the sample is moved through the focus.


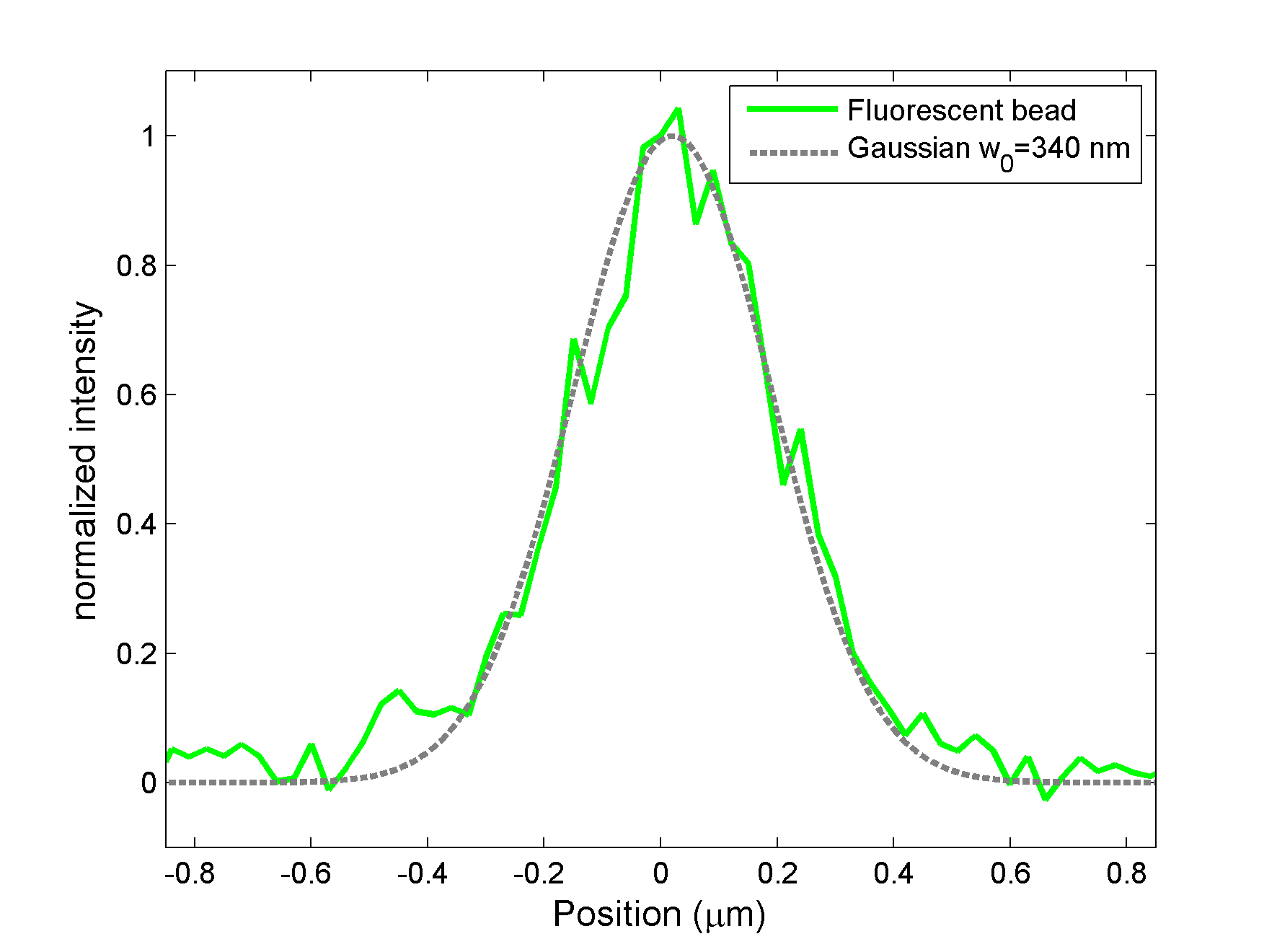


FIG. S2: A cross section through an in focus fluorescent bead (thick green line) taken from Supplementary Movie 2, and a Gaussian with *w_0_*=0.34 μm (thick dashed line). This width corresponds to an illumination spot size of w_0_=295 nm convoluted with the fluorescent bead (diameter 200 nm). The figure illustrates that the beam radius *w_0_* measured with our knife edge method (327±11 nm) is in reasonable agreement with the beam radius derived from measurements on fluorescent beads. The images in Supplementary Movie 2 were acquired using 488 nm excitation and the 40x/0.75 N.A. objective.


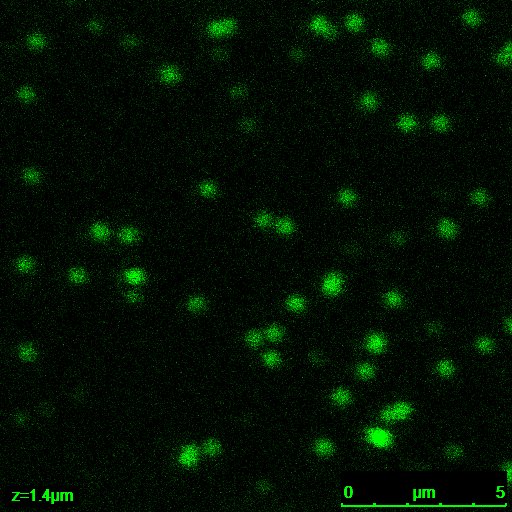


Supplementary Movie 2: A Z-stack of images of 200 nm fluorescent beads (Fluoresbrite, Polysciences inc.) mounted in Vectashield acquired using 488 nm excitation, a detection range of 500-570 nm and the 40x/0.75 N.A. objective. The images are separated with 340 nm in the Z-direction.


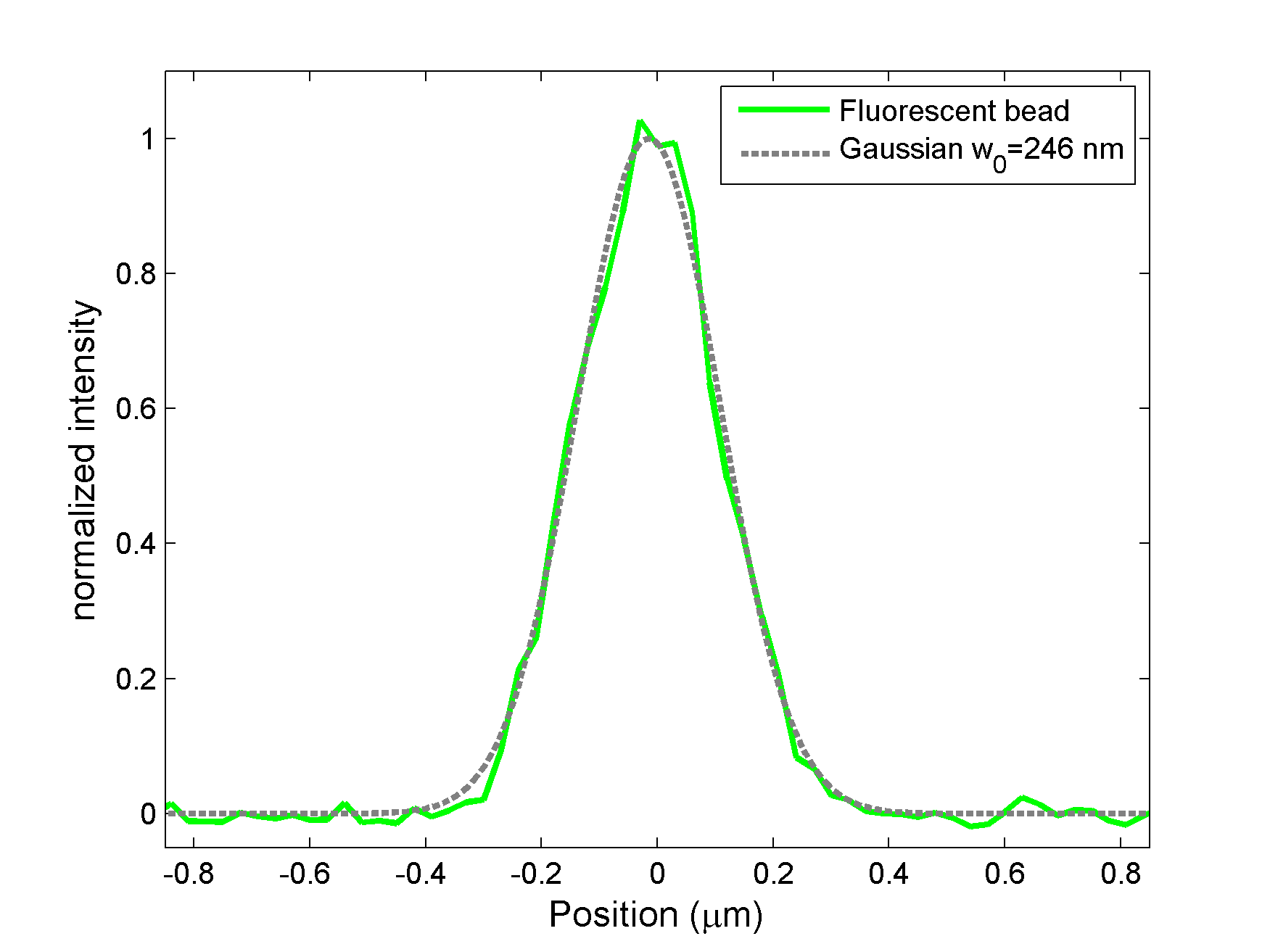


FIG. S3: A cross section through an in focus fluorescent bead (thick green line) taken from Supplementary Movie 3, and a Gaussian with *w_0_*=0.246 μm (thick dashed line). This width corresponds to an illumination spot size of w_0_=178 nm convoluted with the fluorescent bead (diameter 200 nm). The figure illustrates that the beam radius *w_0_* measured with our knife edge method (169±2 nm) is in reasonable agreement with the beam radius derived from measurements on fluorescent beads. The images in Supplementary Movie 2 were acquired using 488 nm excitation and the 40x/1.3 N.A. objective.


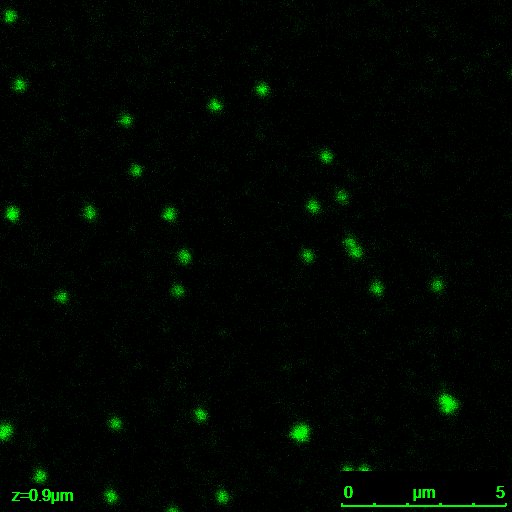


Supplementary Movie 3: A Z-stack of images of 200 nm fluorescent beads (Fluoresbrite, Polysciences inc.) mounted in Vectashield acquired using 488 nm excitation, a detection range of 500-570 nm and the 40x/1.3 N.A. objective. The images are separated with 170 nm in the Z-direction. The astigmatism is clearly visible as the sample is moved through the focus.


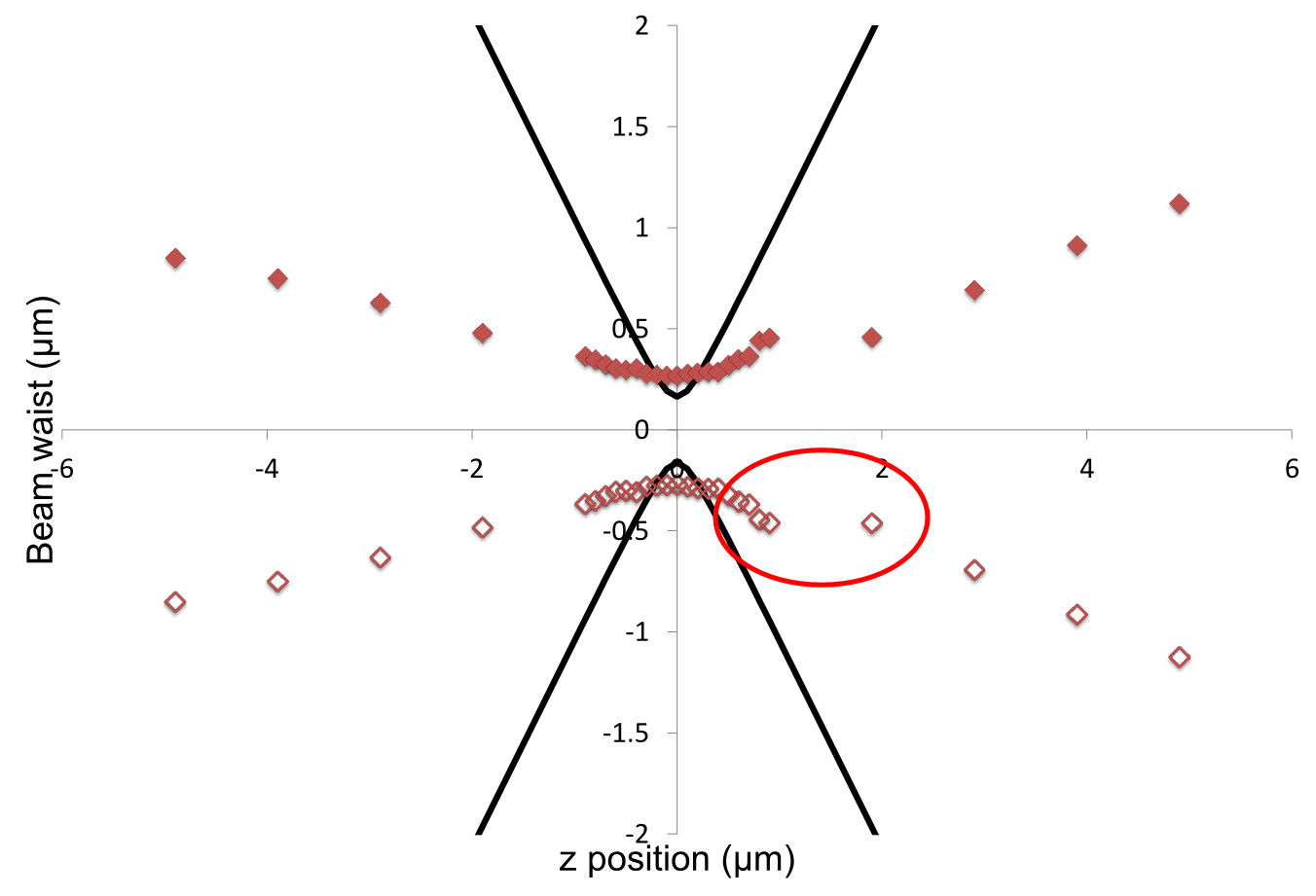


FIG. S4: Experimental data of the evolution of the beam radius for the 40x/1.3 N.A., lens used with a 800 nm laser. The data points (filled diamonds) are presented with the ideal beam propagation for the N.A. of the lens. (thick solid line). The data set below the x axis (open diamonds) is a mirror image of the measured data, to more clearly illustrate the beam propagation. The data illustrates the effect of diffraction from the knife-edge, which results in a small but abrupt change in the beam diameter and a slightly different divergence after the focus.
